# Unveiling Twist Domains in Monolayer MoS_2_ through 4D‐STEM and Unsupervised Machine Learning

**DOI:** 10.1002/smtd.202501065

**Published:** 2025-08-06

**Authors:** Koji Kimoto, Ovidiu Cretu, Koji Harano, Fumihiko Uesugi, Jun Kikkawa, Kohei Aso, Yoshifumi Oshima, Takashi Matsumoto, Yoshiki Sakuma

**Affiliations:** ^1^ Center for Basic Research on Materials National Institute for Materials Science (NIMS) Tsukuba 305‐0047 Japan; ^2^ Research Center for Autonomous Systems Materialogy Institute of Integrated Research Institute of Science Tokyo Yokohama 226‐8501 Japan; ^3^ School of Materials Science Japan Advanced Institute of Science and Technology (JAIST) Nomi 923‐1292 Japan; ^4^ Tokyo Electron Technology Solutions Ltd. Nirasaki 407‐8511 Japan

**Keywords:** 4D STEM, dichalcogenide, metal–organic chemical vapor deposition (MOCVD), MoS_2_, scanning transmission electron microscopy (STEM), unsupervised machine learning

## Abstract

Dichalcogenides, such as molybdenum disulfide (MoS_2_), are being studied extensively due to their 2D feature and various material properties. Although crystal structures are critical for applications, conventional atomic structure analyses have a limited field of view. In this study, the crystal domains of monolayer MoS_2_ synthesized by metal–organic chemical vapor deposition (MOCVD) are analyzed using 4D scanning transmission electron microscopy (STEM) and unsupervised machine learning. Twist domains (±11°) are identified through the nonnegative matrix factorization (NMF) and hierarchical clustering of numerous (>22k) diffraction patterns from a wide field of view. Preprocessing for detecting noncentrosymmetry effectively visualizes the polarities of distinct MoS_2_ domains by highlighting the violation of Friedel's law in diffraction physics. Analyses reveal that the specimen deposited on Al_2_O_3_ (0001) at 850 °C consists of domains measuring ≈100 nm in size and featuring many mirror‐twin boundaries. The findings provide valuable insights into optimizing the MOCVD process and elucidating crystal growth mechanisms.

## Graphical abstract

The twist domains of monolayer MoS_2_ are visualized using 4D scanning transmission electron microscopy and unsupervised machine learning. Numerous (>22k) diffractions are analyzed using nonnegative matrix factorization and hierarchical clustering. Preprocessing to detect noncentrosymmetry clearly visualized the polarity of each MoS_2_ domain based on the violation of Friedel's law.

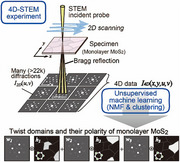

## 1 Introduction

Various applications of dichalcogenide compounds have been investigated,^[^ ^1^, ^2^, ^3^ ^]^ and excellent semiconductor properties are expected, for example, in monolayer MoS_2_ transistors.^[^ ^4^ ^]^ Dichalcogenides have a layered structure, and their atomic structures are directly related to device performance. In particular, electron mobility and optical properties are sensitive to crystal defects and domain structures. Therefore, comprehensive dichalcogenide characterization requires crystal structural analysis at the atomic and submicrometer scales. From the viewpoint of industrial applications, current research efforts are focused on approaches to prepare single‐crystalline films on wafers using various techniques, including metal–organic chemical vapor deposition (MOCVD), instead of the Scotch‐tape method.^[^ ^5^, ^6^ ^]^ Uniformity and scalability are important considerations in the development of deposition technologies for semiconductor devices, and evaluations of multiscale crystalline qualities such as defects, polarity, and grain boundaries over a wide field of view are essential.

Although high‐resolution scanning transmission electron microscopy (STEM) enables the direct observation of the atomic arrangement of MoS_2_, the observable field of view of this technique is limited to a few hundred nanometers.^[^ ^7^ ^]^ Dark‐field TEM can visualize twist domains, although the spatial and angular resolution is limited by the size of the objective aperture (i.e., diffraction limit).

**Figure 1:**
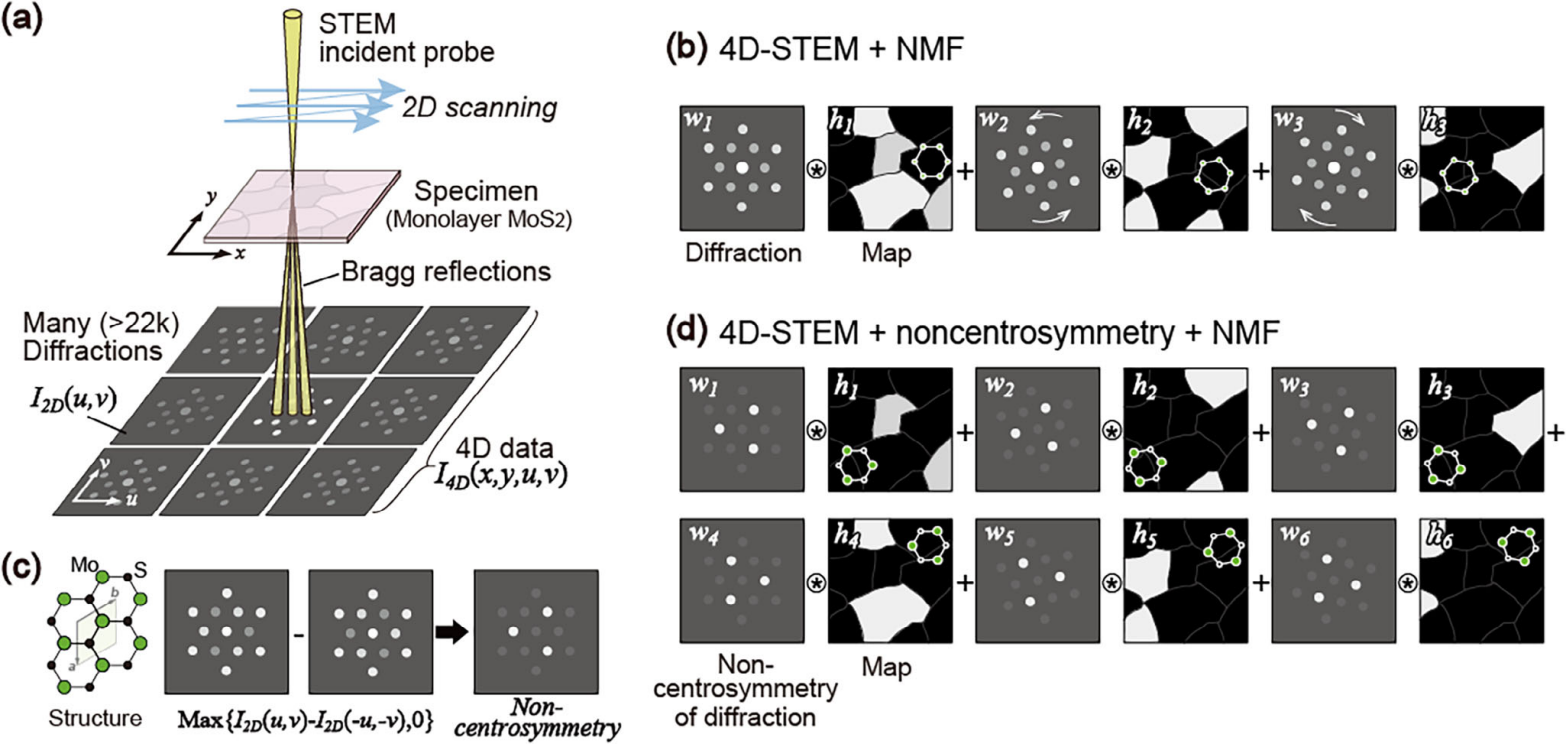
Schematic of 4D‐STEM and nonnegative matrix factorization. a) Experimental configuration of 4D‐STEM. b) Schematic representation of the factorization process for domain analysis. c) Preprocessing for noncentrosymmetry owing to the violation of Friedel's law in the dynamical diffractions. d) Combination of preprocessing and NMF for polarity analysis.

4D STEM^[^ ^8^, ^9^, ^10^, ^11^ ^]^ is a technique used to acquire numerous electron diffractions from nanometer areas under the focus of an incident electron beam; real and reciprocal space information can be constructed from the 4D data as maps and diffractions, respectively (**Figure** **1**a). Recent developments in STEM instrumentation have enabled the acquisition of a large number of diffractions at high speeds; thus, 4D‐STEM has been applied to a wide range of materials.^[^ ^10^ ^]^ While primitive 4D‐STEM could construct virtual dark‐field TEM images, it could not distinguish small‐angle rotation domains of the actual specimens, similar to conventional TEM techniques. Consequently, twist domains and their polarity have not yet been elucidated from a relatively wide field of view.

In this study, multidomain MoS_2_ specimens deposited by MOCVD were analyzed using 4D‐STEM. The numerous (e.g., >22k) diffractions obtained were investigated using diffraction simulations and unsupervised machine learning. Unsupervised machine learning effectively extracts material information from actual experimental data without prior knowledge. In previous studies, we first applied dimensionality reduction by nonnegative matrix factorization (NMF)^[^ ^12^ ^]^ and hierarchical clustering for 4D‐STEM.^[^ ^13^ ^]^ Unlike principal component analysis (PCA), which is another conventional dimensionality‐reduction technique, NMF yields interpretable electron diffractions without the negative peaks produced by PCA. First, we used NMF to factorize the experimental diffractions into a smaller number (e.g., seven) of interpretable diffractions and their corresponding maps (Figure 1b). Based on dynamical diffraction simulations and high‐resolution STEM experiments, we confirmed the directional correspondence between the polarity of monolayer MoS_2_ and its diffraction (Figure 1c). Twist domains consisting of mirror twins were visualized for a wide field of view by NMF with preprocessing to enhance their noncentrosymmetry (Figure 1c,d).

## 2 Results and Discussion

### 2.1 Electron Diffraction Simulation

**Figure 2:**
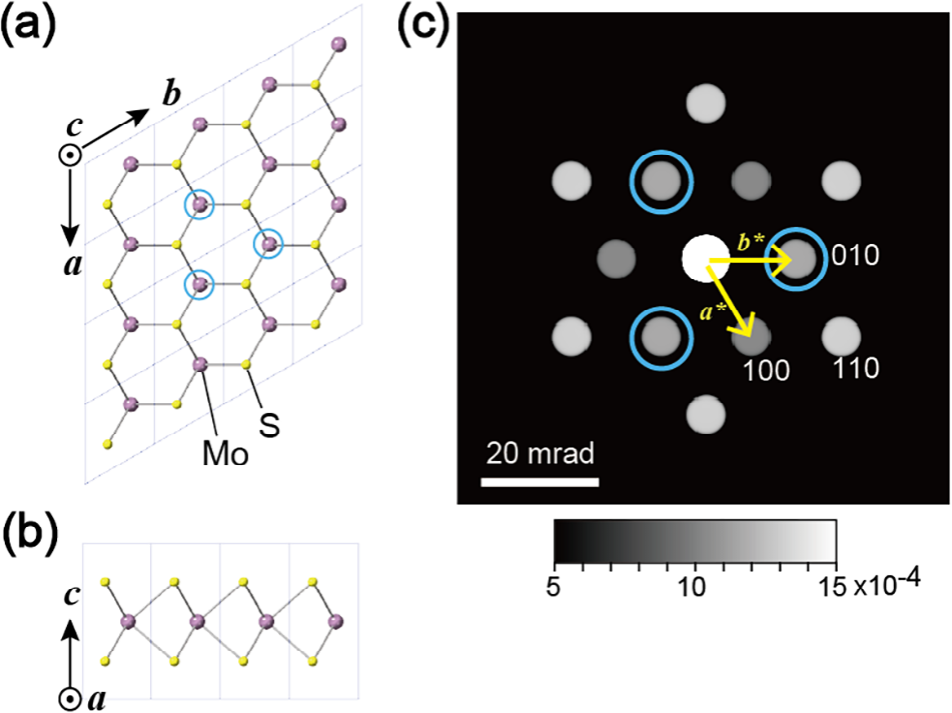
Crystal structure of monolayer MoS_2_ and dynamical diffraction simulation. a, b) Atomic arrangements along the (a) [001] and (b) [100] directions. c) Dynamical calculation of diffraction along the [001] direction.

Unlike X‐ray diffraction, Friedel's law^[^ ^14^ ^]^ is often violated in electron diffraction, even in monolayer MoS_2_, and the noncentrosymmetry of the electron diffractions can be used to determine the polarity of the crystals.^[^ ^11^ ^]^ We confirmed these concepts using both kinematical and dynamical simulations. **Figure** **2**a,b show the crystal structure of monolayer MoS_2_, representing the lower half of the known 2H‐MoS_2_ structure, used in the simulations. CrystalMaker and SingleCrystal (CrystalMaker Software Ltd.)^[^ ^15^ ^]^ were used to calculate the structure factor (i.e., kinematical simulation), and a multislice software (xHREM, HREM Research, Inc.)^[^ ^16^, ^17^ ^]^ was also used for the dynamical simulation, as shown in Figure 2c. In the dynamical diffraction, an intense direct spot at the center and scattered Bragg spots appear as discs owing to the convergent incident probe. Because of the small convergence angle *α* of 3 mrad and the diffraction limit, the size *d* of the incident probe (*d* = 1.22*λ/α, *1.7 nm) is larger than that of the unit cell of the specimen, where the wavelength *λ* of an electron equals to 4.2 pm. In general, complicated intensity modulations (i.e., zero‐order Laue zone patterns) exist in each diffraction disc; however, in the present monolayer case, the intensity within the discs becomes constant.^[^ ^18^ ^]^ The dynamical diffraction intensities of spots 100 and 010 differ, as indicated by the blue open circles reflecting relatively intense spots in Figure 2c. This is the violation of Friedel's law, showing noncentrosymmetry. Details of the kinematical and dynamical intensities are shown in the Table S1 (Supporting Information).

Note the directional correspondence between the polarity of monolayer MoS_2_ (Figure 2a) and the three intense innermost spots (−100, 1–10, 010) (Figure 2c). This relationship in experimental data is often rotated owing to various practical reasons, such as variations in the microscope post‐specimen lens, scanning unit, and camera mechanical/software settings, and the results described in the few reports on rotational correspondence have been inconsistent. To confirm the actual rotational correspondence in our experiment, we observed an atomic‐resolution ADF image and a diffraction pattern of the same area, and confirmed the directional correspondence. In the present experimental configuration, the correspondence of the diffractions acquired using the energy filter (Figure 1c) was found to be rotated by 180° compared with the simulated results (Figure 2a,c). Hence, we analyzed the polarities at each position based on the confirmed correspondence; the details and experimental results of this directional correspondence are described in the Supporting Information (Figure S1, Supporting Information).

### 2.2 Conventional 4D‐STEM Analysis

**Figure 3:**
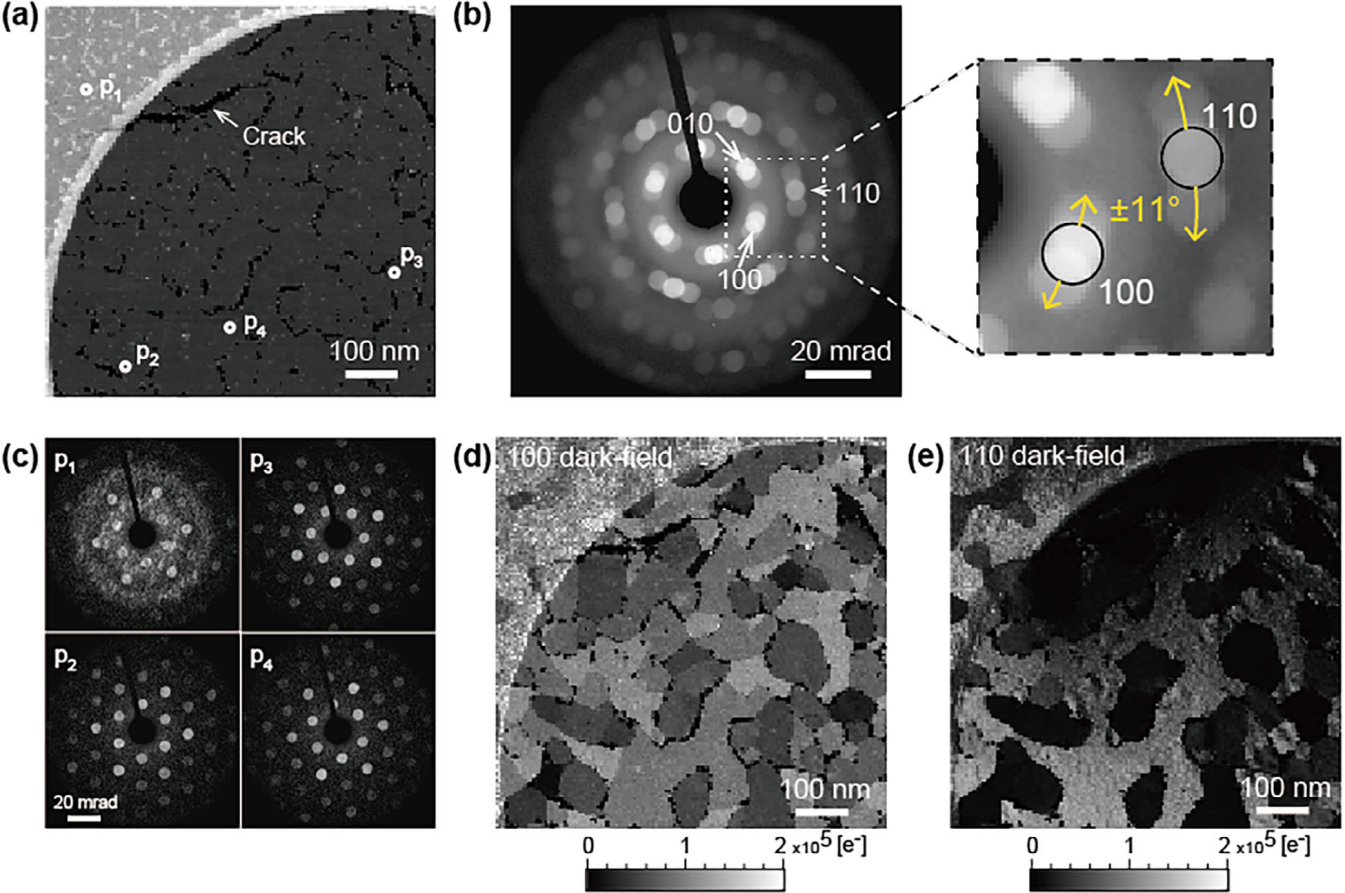
Conventional 4D‐STEM results of the MoS_2_ layer. a) ADF image. b) Integrated diffraction from the entire field of view and enlarged diffraction. The open circles indicate detection areas selected for virtual dark‐field imaging. c) Examples of diffractions at four points. d, e) Virtual dark‐field images of spots (d) 100 and (e) 110.

**Figure** **3** shows the conventional 4D‐STEM analyses of the MoS_2_ specimen. The ADF image (Figure 3a) shows an amorphous carbon film of Quantifoil (upper left corner) and the MoS_2_ film, which has several cracks. The ADF image shows nearly uniform intensity, suggesting a monolayer of MoS_2_, although crystalline domains are not visualized. The diffraction pattern integrated from the entire field of view is shown in Figure 3b. Each diffraction spot is split into three directions, although the polarity could not be identified due to the integration. Figure 3c shows examples of diffractions observed at four points (***p**_**1**_*, ***p**_**2**_*, ***p**_**3**_*, ***p**_**4**_*). The diffraction at ***p**_**1**_* reveals the amorphous rings of the carbon film and crystalline spots. Because MoS_2_ is in monolayer form, its diffraction intensity is comparable with that of the carbon film. The diffractions at positions ***p**_**2**_*, ***p**_**3**_*, and ***p**_**4**_* show single‐crystalline spots, with each direction rotating slightly. The rotation angle is ±11°, and the rotated innermost spots are overlapped, as shown in Figure 3b.

Virtual dark‐field imaging, a conventional 4D‐STEM technique, was conducted, and the images were constructed by selecting part of the diffractions from the acquired 4D data. Figure 3d,e show the virtual dark‐field images of spots 100 and 110, which are indicated by the open circles in the magnified diffraction in Figure 3b. In the dark‐field image of spot 100 (Figure 3d), all domains are bright because of overlapping, rendering the clarification of each domain complicated. By contrast, the domains in spot 110 are separated and could be clearly identified (Figure 3e). The second‐innermost spots, such as spot 110, cannot be used for polarity determination, as shown in Table S1 (Supporting Information). In addition, the intensity of spot 110 changes sensitively owing to the specimen tilt (e.g., the dark areas near the crack in Figure 3e). The effect of specimen tilt (i.e., bending) was confirmed by multislice simulations, as discussed in the Figure S2 (Supporting Information). Although the intensities of the six innermost spots did not change, those of the second‐innermost spots changed significantly, even with a small tilt of <10°. Note that the problems of intensity variation owing to the specimen tilt and diffraction spot overlap are not unique to 4D‐STEM; they are also encountered in general TEM techniques, including dark‐field TEM.

### 2.3 Dimensionality Reduction by NMF and Hierarchical Clustering

**Figure 4:**
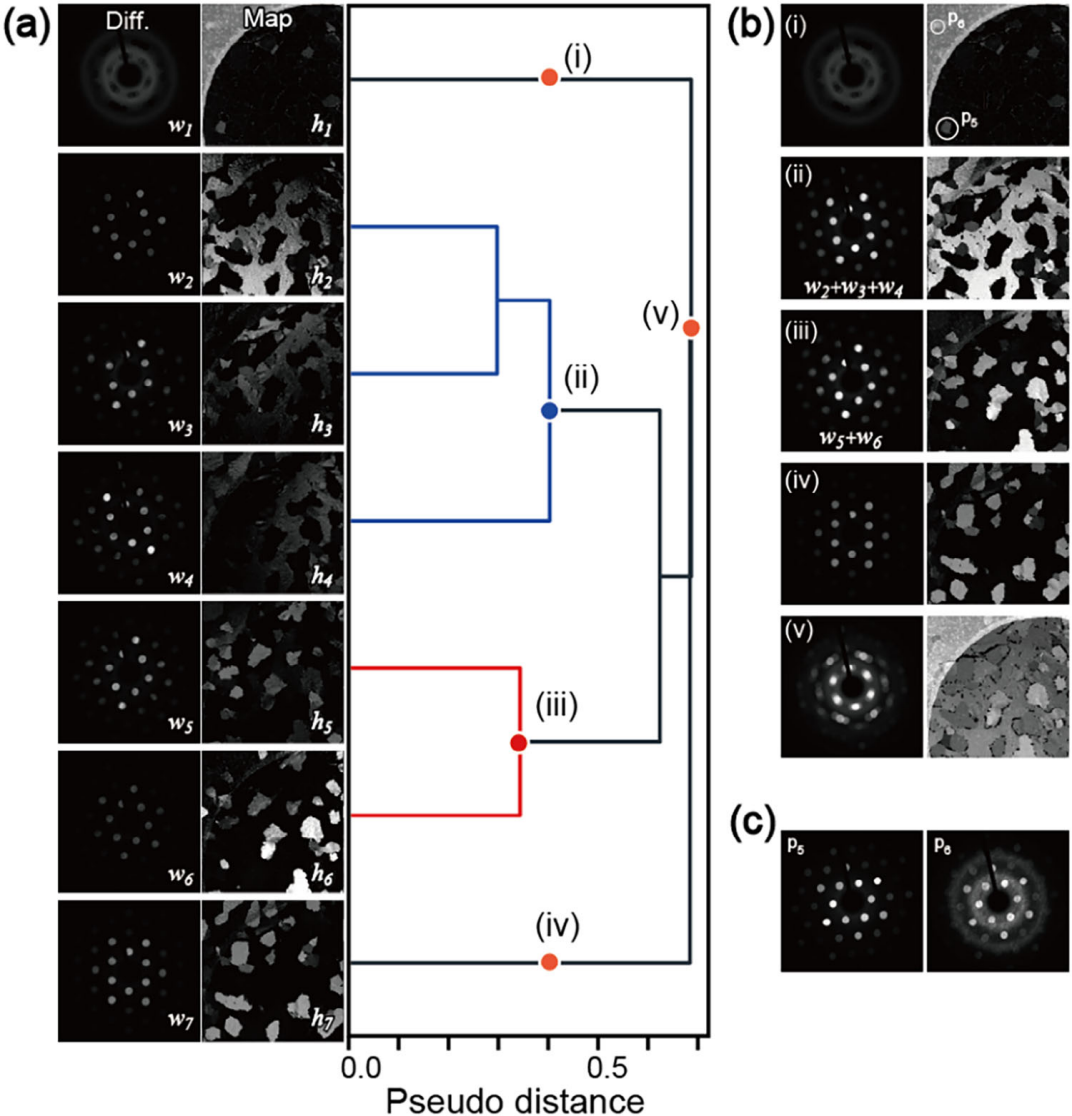
NMF and hierarchical clustering of the 4D‐STEM results. a) Seven pairs of factorized diffractions and maps and the dendrogram of hierarchical clustering. b) Integration of the diffractions and maps using the clustering. c) Experimental diffractions at ***p_5_*** and ***p_6_***, which are minority components neglected in the integrated diffraction and NMF.

Based on the integrated diffraction shown in Figure 3b, we first attempted to factorize the experimental many (>22k) diffractions into seven components consisting of one amorphous and three pairs of crystalline mirror domains in this field of view. The details of NMF for 4D‐STEM are provided in the Experimental Section. **Figure** **4**a shows the seven pairs of diffractions and the corresponding maps obtained using NMF. The first component ***w**_**1**_* appears to be amorphous. The remaining six crystalline components (***w**_**2**_* – ***w**_**7**_*) show centrosymmetric diffractions, indicating intensity changes in the second‐innermost spots owing to specimen tilting. Thus, NMF alone cannot effectively discriminate polarity from the actual experimental results. Figure 4a also shows a dendrogram obtained by hierarchical clustering based on the similarity of the seven diffractions. The horizontal axis represents the similarity (pseudo‐distance) between diffractions, which is calculated as the correlation coefficient. According to the dendrogram, the seven components can be categorized into four groups as follows: (i) amorphous ***w**_**1**_*; (ii) crystalline ***w**_**2**_*, ***w**_**3**_*, and ***w**_**4**_*; (ii) crystalline ***w**_**5**_* and ***w**_**6**_*; and (iv) crystalline ***w**_**7**_*; however, the major component is the crystalline domains (ii). Each integrated diffraction and map is shown in Figure 4b. Thus, hierarchical clustering is effective in regrouping the components and correcting the effect of specimen tilting on the experimental results.

However, these analyses fail to separate mirror domains with different polarities because the intensity variation owing to specimen tilting is larger than the difference (≈12%) expected from the violation of Friedel's law. This issue can be solved by appropriate preprocessing, as described in Section 2.4.

A few bright domains, as indicated by ***p_5_*** and ***p_6_***, can be observed in map (i), which is identical to the amorphous map ***h_1_***. Figure 4c shows the diffractions of regions ***p_5_*** and ***p_6_***; in these regions, other domains with different orientations can be observed as minority components. These domains could not be detected by integrated diffraction (Figure 3b) owing to their weak intensity. Although these domains could not be resolved by NMF with a seven‐component assumption, their minor components can be observed using both maps and diffractions, as demonstrated in this study.

### 2.4 Polarity Mapping Using Preprocessed Diffractions and 4D‐STEM

We apply appropriate preprocessing to mitigate the effects of specimen tilting and analyze the domains, including their polarities. Some preprocessing techniques, such as cepstrum, have already been reported to deduce diffraction symmetry^[^ ^19^ ^]^ thereby avoiding the effect of specimen tilting. However, because the cepstrum includes a modulus procedure after the Fourier transform, the cepstrum always becomes centrosymmetric, prohibiting the detection of material polarity. Hence, we applied different preprocessing strategies to detect deviations from the central symmetry.

**Figure 5:**
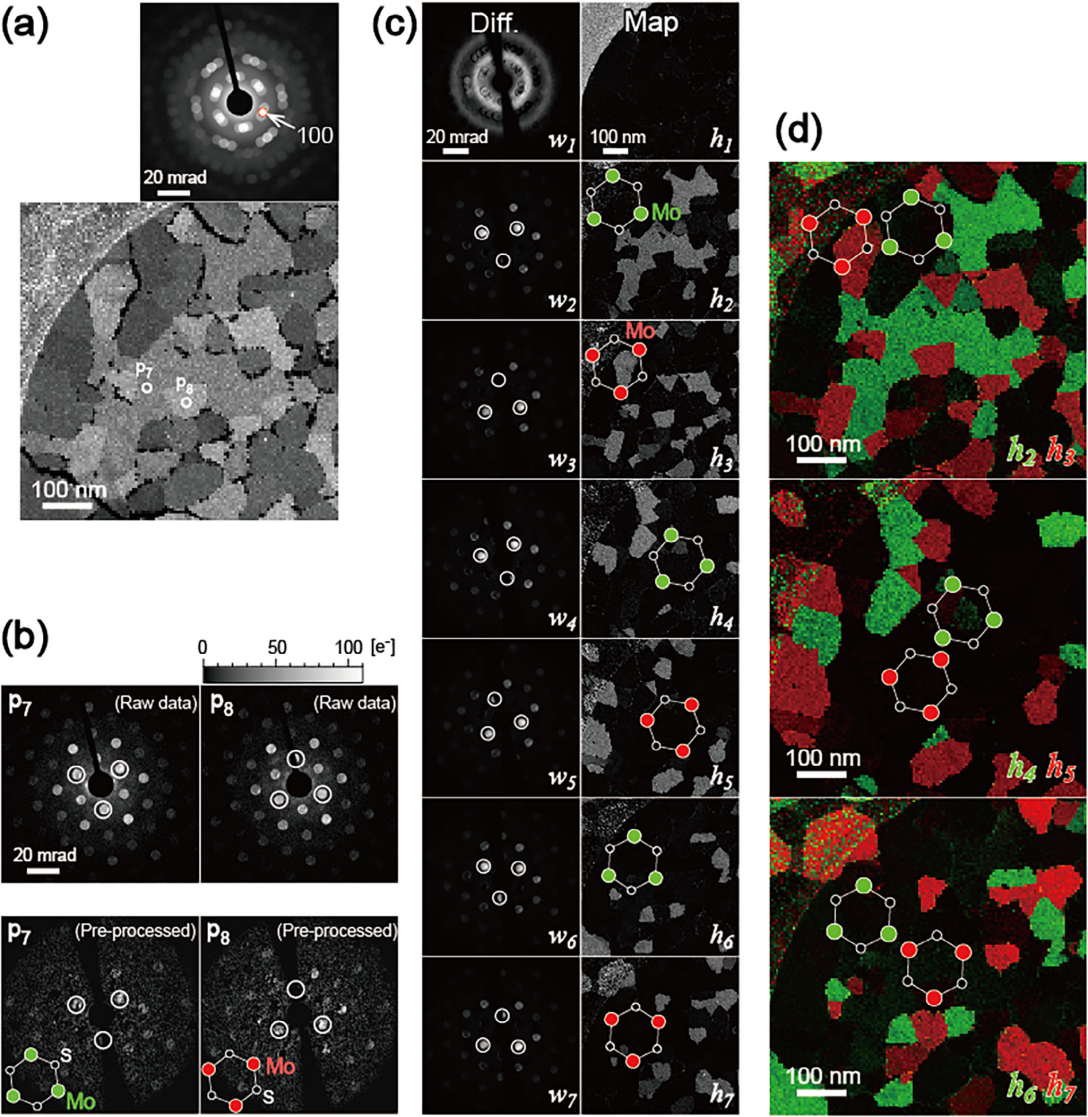
Polarity analysis of MoS_2_. a) Integrated diffraction and virtual dark‐field images of spot 100. b) Experimental diffractions at positions ***p**_**7**_* and ***p_8_***, and the corresponding pre‐processed diffraction for detecting noncentrosymmetry. The white circles indicate the positions of the six innermost spots with strong diffraction. c) NMF results based on the preprocessed data. d) Images colored by the polarity of each of the three orientations based on the NMF results.

**Figure** **5**a shows the integrated diffraction and a virtual dark‐field image of spot 100. The diffractions at positions ***p_7_*** and ***p_8_*** are shown in Figure 5b, with the three innermost spots showing the strongest intensity indicated by white open circles. The brightness of each image corresponds to the number of electrons, as indicated by the brightness bar. The polarity in real space, which was rotated by 180° compared with that in Figure 2b, as mentioned earlier, is shown as insets.

The noncentrosymmetry of each diffraction was calculated as a positive value by subtracting the diffractions rotated by 180° using the following equation:

(1) $$\mathrm{Noncentrosymmetry}=\max(I_{2D}\left(u,v\right)-I_{2D}\left(-u,-v\right),0)$$

where ***I*** *_2D_* (*u*,*v*) is the experimental diffraction in the reciprocal coordinates (*u,v*). Part of the beam stopper and its symmetrical area were masked before the preprocessing. The processed diffractions at ***p_7_*** and ***p_8_*** are shown in the lower part of Figure 5b. This preprocessing step transformed all (>22k) diffractions of the experimental 4D data. Noncentrosymmetric preprocessing is susceptible to diffraction off‐centering (i.e., the origins of *u* and *v*) because of the steep intensity changes at the edges of the diffraction discs. In this study, the center of the diffraction was determined with subpixel accuracy from the cross‐correlation of the integrated diffraction.

As shown in Figure 5b, the preprocessed diffractions appear to be noisy. The intensity for the innermost spots obtained in the experiment was ≈100 electrons per pixel. Because quantum noise follows a Poisson distribution, the expected quantum noise‐to‐signal ratio (√*n*/*n*) of *n* electrons is slightly smaller than the difference between that of spots 100 and 010 (≈12%), as listed on Table S1 (Supporting Information). In other words, the experimental setup was optimized to obtain the required signals within the shortest possible time with the minimum electron dose possible.

Figure 5c shows the results of dimensionality reduction using NMF, assuming seven components. One amorphous component and six crystalline components with different polarities were successfully factorized. Although amorphous diffraction (***w_1_***) must be eliminated because of its centrosymmetry, the intensity residuals owing to Poisson noise exhibit halo rings. Crystalline diffraction (***w_2_***–***w_7_***) was used to determine the crystal orientation and polarity, as indicated by the white circles and each inset, respectively. The factorized diffractions (***w_2_***–***w_7_***) have a higher signal‐to‐noise ratio than the individual preprocessed diffractions, and their polarities can be easily determined. Figure 5d visualizes these results by showing a pseudocolor image of the three orientations, with the polarity reversed for each orientation. The mirror domains are often adjacent to each other, and the grain boundaries are very complex. Localized polarity inversion has been reported when using atomic‐resolution STEM; however, it has not been previously visualized for wide‐area measurements. In particular, this study allows for statistical observations of unique cases such as complicated multidomain specimens. These results are expected to provide useful information for the future development of single‐domain growth through MOCVD optimization.

## 3 Conclusion

In this study, we analyzed the twist domains of monolayer MoS_2_ using dynamical electron diffraction, 4D‐STEM, and unsupervised machine learning techniques, and discussed the advantages of these combinations. Compared to conventional dark‐field TEM imaging, the 4D‐STEM study not only exhibits superior spatial/angular resolution but also demonstrates robustness against specimen tilting and dose efficiency. A detailed comparison of the two methods is provided in the supporting information section.

While virtual dark‐field imaging by conventional 4D‐STEM allows for the versatile selection of spots, it cannot resolve overlapped diffraction spots. In this study, we demonstrated that NMF enables the factorization of these overlapped spots into their correct positions. Moreover, preprocessing for noncentrosymmetry is highly effective in separating the effects of polarity and specimen tilt.

Notably, conventional 4D‐STEM is insufficient for visualizing the mirror‐twin boundary of monolayer MoS_2_. Advanced material characterization with effective physics‐informed machine learning techniques can be established by integrating knowledge from electron microscopy, diffraction crystallography, and materials science. This method can be applied to other dichalcogenides and single‐ or few‐layer 2D materials.

## 4 Experimental Section

### Specimen Preparation

A MoS_2_ monolayer was deposited on an Al_2_O_3_ (0001) substrate at a temperature of 850 °C by MOCVD. The source gases utilized for MOCVD were MoO_2_Cl_2_ and H_2_S, with N_2_ as the carrier gas. Details of the deposition process have been reported elsewhere.^[^ ^6^ ^]^ Although the authors had already optimized the growth conditions for synthesizing micrometer‐sized single‐crystalline MoS_2_, multidomain specimens were analyzed for material characterization in the present study. Subsequently, the MoS_2_ layer was peeled off from the Al_2_O_3_ substrate using polymethyl methacrylate^[^ ^20^ ^]^ and transferred to a holey carbon film for STEM observations (Quantifoil, Quantifoil Micro Tools GmbH). To prevent contamination during STEM observations, the transferred specimen was cleaned by UV irradiation in an O_2_ atmosphere^[^ ^21^ ^]^ for 25 min. Although the specimen underwent partial etching and cracking owing to UV irradiation, there was no atomic defect as observed by an atomic‐resolution STEM image (Figure S1a, Supporting Information)

### 4D‐STEM Measurements

The 4D‐STEM measurements were conducted using an aberration‐corrected scanning transmission electron microscope (Titan Cubed, Thermo Fisher Scientific) at an acceleration voltage of 80 kV. The convergence semi‐angles for 4D‐STEM and atomic‐resolution STEM were 3 and 25 mrad, respectively. The descan function of the microscope, which was carefully adjusted by a Thermo Fisher Scientific engineer, stabilized the positions of the diffraction spots during scanning. A custom‐made ϕ 20 micrometer aperture (Daiwa Techno Systems) was utilized for 4D‐STEM, the circularity of which was important for the preprocessing of noncentrosymmetry. Diffraction patterns were acquired using an energy‐filtered camera (Continuum HR, Gatan Inc.)^[^ ^22^ ^]^ with an energy width of 20 eV. The conversion efficiency (58 counts per electron) of the energy‐filtered camera was experimentally calibrated, and the detected counts were converted into numbers of electrons. To avoid afterglow artifacts (see Figure S1b, Supporting Information) owing to spots with intense transmission (i.e., 1000 times higher than that of the diffracted spots of monolayer MoS_2_), a beam stopper was inserted to acquire the diffractions. ADF images were obtained using a Gatan HAADF detector (Model 806, Gatan Inc.). The 4D measurement conditions were set to 150 × 150 points in real space and 256 × 256 pixels in reciprocal space. The exposure time for each diffraction was set to 50 ms for Figure 3 and 100 ms for Figure 5. The 4D‐STEM data were clipped (228 × 228) to center the diffraction patterns after acquisition.

### Overview of NMF for 4D‐STEM

The combination of machine learning and STEM has attracted considerable attention.^[^ ^23^, ^24^ ^]^ Dimensionality reduction using NMF was a representative method of unsupervised machine learning. Here, a brief overview of NMF^[^ ^25^, ^26^, ^27^ ^]^ and its combination with 4D‐STEM was provided. The experimental data, matrix ***X***, were approximated by the product of the lower‐rank matrices ***W*** and ***H*** consisting of nonnegative elements:

(2) X≅WH

where ***W*** and ***H*** denote the basis and its coefficient, respectively. The 4D data were transformed into a 2D matrix (i.e., unfolding) for matrix calculation. In this study, the rows and columns of ***X*** represent the reciprocal and real‐space coordinates of 4D‐STEM, respectively. The column vectors of matrix ***W*** and row vectors of matrix ***H*** represent the diffractions and corresponding maps, respectively. The low‐rank matrices ***W*** and ***H*** were determined by minimizing a cost function *D* based on the Frobenius norm ||·||*_F_* of the error as follows:

(3) $$D\left(X∥\mathrm{WH}\right)=\frac{1}{2}{{\left\|X-\mathrm{WH}\right\|}^{2}}_{F}$$

Equation (3) can be minimized in several ways. Here, the alternating least‐squares (ALS) algorithm was employed, also called coordinate descent. The ALS algorithm was applied using the following equations:

(4) $$W\leftarrow {\left[\left(XH^{T}\right){\left(HH^{T}\right)}^{-1}\right]}_{+}$$

(5) $$H\leftarrow {\left[{\left(W^{T}W\right)}^{-1}\left(W^{T}X\right)\right]}_{+}$$

The symbol [·]_+_ represents a nonnegativity constraint projection,^[^ ^26^ ^]^ which was defined as [W]+=max(W,0). Finally, the converged matrices ***W*** and ***H*** were transformed into diffraction ***w_k_*** (*u,v*) and map ***h_k_*** (*x,y*) (*k* = 1,2,…) pairs (i.e., refolding).

In general, the number of components, which was the columns of ***W*** and the rows in ***H***, was unknown; however, it can be estimated to be seven based on the present experimental diffraction (Figure 3b) and the MoS_2_ crystal structure (Figure 2a,b). Convergence of the iterations of Equations (4) and (5) can be determined by the tolerance^[^ ^28^ ^]^ or monitoring the mean square errors.^[^ ^12^, ^13^ ^]^ The possibility of convergence to a local minimum was a known feature of NMF, and a sufficiently reliable global minimum can be found by performing multiple calculations and comparing the mean square errors. Further details on NMF and its combination with 4D‐STEM have been reported elsewhere.^[^ ^12^, ^13^ ^]^

In the present study, DigitalMicrograph software (Gatan Inc.) was used to acquire and analyze the experimental 4D data.^[^ ^29^ ^]^ The various preprocessing steps (e.g., noncentrosymmetry calculations) were performed using in‐house DigitalMicrograph scripts. Python on DigitalMicrograph was also utilized for unsupervised machine learning, e.g., Scikit‐Learn,^[^ ^30^ ^]^ NumPy,^[^ ^31^ ^]^ SciPy,^[^ ^32^ ^]^ and MatPlotLib.^[^ ^33^ ^]^ Hierarchical clustering was performed by calculating the similarity of diffractions using cross‐correlation to obtain a pseudo‐distance. These calculations were also carried out using in‐house scripts on DigitalMicrograph. Examples of DigitalMicrograph scripts have been reported elsewhere.^[^ ^12^, ^13^, ^34^ ^]^

The computational cost was a drawback of NMF because iterative matrix calculations of Equation (4) and (5) were required. The required number of iterations may range from tens to hundreds (e.g., ≈100 in our previous study).^[^ ^12^ ^]^ Due to the large 4D‐STEM dataset, the computational cost was high. The 4D‐STEM data in this study were calculated using a desktop computer with an AMD Ryzen 9 9950X. Assuming seven components as shown in Figures 3 and 5, 100 iterations for 4D‐STEM data (4.6 GB, 150 × 150 × 228 × 228) took ≈1 min.

## Conflict of Interest

The authors declare no conflict of interest.

## Supporting information



Supporting Information

## Data Availability

The data that support the findings of this study are available from the corresponding author upon reasonable request.
