# Supplementary material for: Unveiling Twist Domains in Monolayer MoS2 through 4D‐STEM and Unsupervised Machine Learning
Source: Small Methods. 2025 Aug 6;9(9):e01065. doi: 10.1002/smtd.202501065 (PMC12464660; doi:10.1002/smtd.202501065)
Supplement: Supplementary file 1 — Supporting Information [file SMTD-9-e01065-s001.docx]

# [Supporting Information]

# Unveiling Twist Domains in Monolayer MoS_2_ Through 4D-STEM and Unsupervised Machine Learning

*Koji Kimoto*, Ovidiu Cretu, Koji Harano, Fumihiko Uesugi, Jun Kikkawa, Kohei Aso, Yoshifumi Oshima, Takashi Matsumoto, and Yoshiki Sakuma*

# 1. Calculated Intensity Ratios of Bragg Spots

We calculated the electron diffractions of monolayer MoS₂ using kinematical and dynamical simulations. CrystalMaker and SingleCrystal (CrystalMaker Software Ltd.)^[15]^ were used to calculate the structure factor *F* (i.e., kinematical simulation), and a multislice software (xHREM, HREM Research, Inc.)^[16, 17]^ was also used for the dynamical simulation. Here, we demonstrate that the violation of the Friedel's law for determining polarity can be simulated using dynamical simulation.

**Table S1** shows the scattering intensities of the innermost and second-innermost Bragg spots in both the kinematical and dynamical simulations. Although the intensities of the six innermost Bragg spots, 100, -110, 0-10, -100, 1-10, and 010, are equal in the kinematical simulation, spots -100, 1-10, and 010 show 12% greater intensity in the dynamical simulation, and it is in agreement with the results of previous studies. The diffractions of the second-innermost spots, 110, -210, 1-20, -1-10, 2-10, and -120, were stronger than those of the innermost spots; however, their intensities were identical, that is, independent of the polarity of the material.

**Table S1**. Kinematical and dynamical diffraction simulations of monolayer MoS_2_

| Reflection indices | Kinematical calculation | | Dynamical simulation | |
| --- | --- | --- | --- | --- |
| *hkl* | \|*F_hkl_*\|^2^ | Ratio | Intensity | Ratio |
| 100, -110, 0-10 | 36.4 | 1 | 0.001036 | 1 |
| -100, 1-10, 010 | 36.4 | 1.00 | 0.001163 | 1.12 |
| 110, -210, 1-20 | 47.1 | 1.29 | 0.001318 | 1.27 |
| -1-10, 2-10, -120 | 47.1 | 1.29 | 0.001318 | 1.27 |

# 2. Directional Correspondence Between STEM Images and Diffraction Patterns

The directional correspondence between STEM/TEM images and diffraction patterns may change for various reasons, such as alterations in the mechanical installation and software settings. To confirm their actual correspondence, we observed the atomic-resolution ADF images and nanobeam diffraction patterns of the same specimen area using an aberration-corrected microscope (Titan-Cubed, Thermo Fisher Scientific) at an acceleration voltage of 80 kV. The convergence angle was varied from 25 (ADF imaging) to 3 (4D-STEM) mrad by changing the condenser aperture and lens settings.

**Figure S1a** shows an atomic-resolution ADF image of monolayer MoS_2_. The specimen was mechanically aligned such that the zigzag direction was oriented horizontally. The atomic arrangements of Mo and S atoms in the ADF image were identified based on their contrast, thereby enabling the determination of their polarity. It should be noted that there is no atomic defect due to specimen preparation, including UV-O_3_ cleaning. Subsequently, the convergence angle was set to 3 mrad, and nanobeam diffraction was conducted using an energy-filtered system under the same conditions used for 4D-STEM, except for the beam stopper. Artifacts were observed on both sides of the intense direct spot (arrows in **Figure S1b**); therefore, a beam stopper was employed in the 4D-STEM experiments in this study. From the six innermost spots, three spots with strong diffraction intensity were indicated by the open circles. The intensity ratios of these selected spots relative to the direct spot was 9.2×10^-4^, while that of the spots with weaker diffraction was 7.8×10^-4^. The difference in these intensities prove that the specimen is indeed single-layer MoS₂. A comparison of the arrangement of Mo in real space with the spots in weak space reveals a 180° rotation compared with that in **Figure 1.** Thus, we analyzed the multidomain specimens using energy-filtered diffraction and confirmed the actual directional correspondence between STEM/TEM images and diffraction patterns, as shown in **Figure S1a and S1b**.


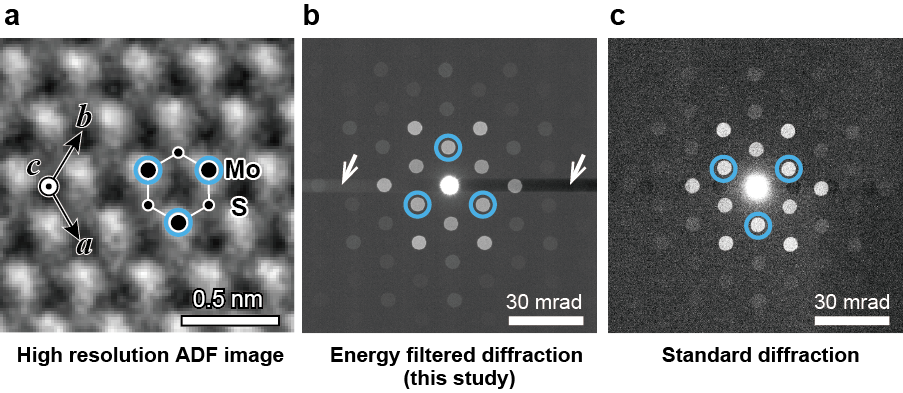


**Figure S1.** **Directional correspondence between actual STEM image and diffraction patterns. (a)** Annular dark-field image. **(b)** Example of the energy-filtered diffractions utilized in this study. **(c)** Example of a conventional electron diffraction acquired using other cameras mounted on the microscope column.

Electron diffraction patterns were also obtained using a standard camera (OneView, Gatan Inc.) mounted on the microscope column. This experimental configuration is frequently used in numerous TEM/STEM studies, and the results are shown in **Figure S1c**. For this case, the orientation of the innermost spots with the strongest intensity was identical to the simulation as in **Figure 1**. In other words, the energy filter pattern was rotated by 180°.

# 3. Changes in Diffraction Intensity and Centrosymmetry Owing to Specimen Tilting

The intensity and symmetry of a diffraction can be altered by varying the specimen tilt. In this study, we investigated the effect of specimen tilt on the structural analysis using dynamical simulations. We used a multislice simulation (xHREM, HREM Research Inc.) that accurately treats specimen tilting. We tilted the direction of incidence by 1°–16° relative to MoS_2_ [001] along two directions and examined the change in diffraction intensity and centrosymmetry, particularly for the six innermost spots (100, -110, 0-10, 010, -100, and 1-10), which were used for polarity determination, and the six second-innermost spots showing the strongest intensity.

The on-axis simulation is shown in **Figure S2a**; here, the top image shows the diffraction of the specimen, while the bottom image shows its noncentrosymmetry. The diffraction intensity was defined as the ratio of the incident intensity. Noncentrosymmetry was calculated as $max(\boldsymbol{I}_{\boldsymbol{2}\boldsymbol{D}}\left( u,v \right)-\boldsymbol{I}_{\boldsymbol{2}\boldsymbol{D}}\left( -u,-v \right),0)$. As mentioned in the main text, the intensities of the six innermost spots differed because of the violation of Friedel’s law, which can be used to evaluate the polarity of MoS_2_. The intensity of the second-innermost spots shows central symmetry, as indicated by the circles.

The results of tilting along the [120] (armchair direction) and [100] (zigzag direction) directions (**Figure S2b**) are shown in **Figure S2c** and **S2d**, respectively. The yellow arrows in the diffraction patterns indicate the tilt direction. The innermost spots showed the same intensity difference regardless of the tilting angle, and their polarities could be determined even when the specimen is tilted. On the other hand, the second-innermost spots lost their central symmetry owing to tilting. The intensity of the higher-order reflections changed significantly when the sample was tilted by less than 10°. Hence, if preparing a flat sample is not possible, analysis using clustering or other similar methods is necessary, as described in the main text.


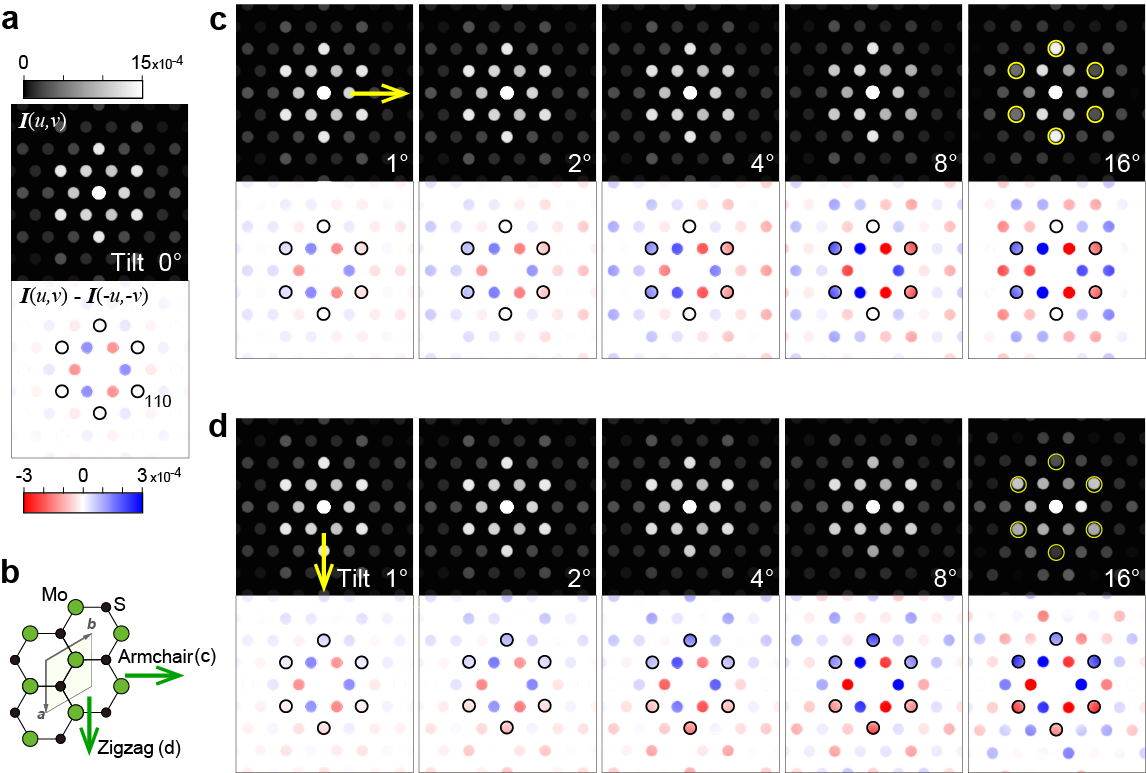


**Figure S2.** **Dynamical diffraction simulation of a tilted specimen. (a)** On-axial diffraction and its noncentrosymmetry. **(b)** Atomic arrangement and tilting direction. **(c)** Diffractions tilted in the chair direction. **(d)** Diffractions tilted in the zigzag direction.

# 4. Accuracy on Polarity Mapping

As shown in the main text, there are some inaccuracies in the polarity mapping in Figure 5d. There are two main reasons for these inaccuracies.

First, there is a fundamental issue with the NMF algorithm. NMF is known to be unable to identify the number of elements. Therefore, in NMF cases where there are minority domains whose existence does not appear in the integral diffraction pattern, it is possible that they cannot be separated by NMF (as with the domains at p5 and p6 in Figure 4b). The determination of the number of elements in NMF is still under discussion in unsupervised machine learning.

The other reason is an experimental issue: quantum noise, which has been discussed in the main text. Quantum noise has a significant effect on the map of the carbon film (see the upper left corner of Figure 5d). This is because the expected quantum noise level from the number of electrons is the same as the noncentrosymmetry signal level (10%). Noise can be reduced by elongating the exposure time (0.1 s in Figure 5) or by assuming crystallographic symmetry in the diffraction. We are currently developing software for this purpose.

# 5. Comparison with conventional dark-field TEM observations

Here, we compare dark-field TEM (DF-TEM), which is an established method, with the combination of 4D-STEM and machine learning used in this study from the following five perspectives.

a) Spatial resolution

In DF-TEM, spatial resolution deteriorates significantly when measurements are taken with a small objective aperture to improve angular resolution. As is well known, spatial resolution d and angular resolution *α* are inversely proportional to each other, *d*∝1/*α*. In 4D-STEM, increasing the convergence angle is also necessary to construct a fine probe; however, as demonstrated in this study, NMF can separate overlapping diffraction discs. In the current 4D-STEM setup, the convergence angle *α* was 3 mrad and the probe diameter was 1.7 nm, but in principle, further improvements are possible.

b) Angular resolution

In DF-TEM, rotation cannot be detected as long as twisted Bragg spots of a few degrees are within the range of the objective aperture. By contrast, 4D-STEM can measure twist angles with relatively high precision. For example, rotations of less than one degree can be distinguished if there are reflections with large scattering angles.

c) Total acquisition time

In DF-TEM, the acquisition time for a single TEM image is short, but when multiple twist domains are present, many TEM images must be acquired. For instance, the specimen examined in this study exhibited three twist angles in integrated diffraction, necessitating at least six TEM images (three for 100 reflections and three for -100). On the other hand, in 4D-STEM, the total acquisition time increases proportionally to the number of points in real space. This long acquisition time is a major disadvantage of 4D-STEM, although the development of high-speed detectors is underway. In this study, the diffraction acquisition time for one point was 0.05–0.1 seconds; however, a new detector capable of acquiring images in less than 1 ms is currently under development.

Furthermore, we would like to point out some practical issues that were not mentioned in the above comments.

d) Robustness against specimen tilt

When the actual specimen is bent, the intensity of the DF-TEM image changes as shown in Figure 3(e). It is difficult to distinguish whether the change in intensity is due to a change in polarity or due to bending. In contrast, 4D-STEM measures all diffraction intensities, enabling determination of whether the specimen is bent at each position. Additionally, as demonstrated in this study, 4D-STEM enables analysis corrected for specimen bending via unsupervised machine learning (i.e., hierarchical clustering).

e) Total electron dose

In 4D-STEM, all electrons irradiated at each point are detected. In contrast, DF-TEM does not detect electrons outside the objective aperture. To measure the twist angle, multiple DF images are required (at least six in this specimen), resulting in a larger number of electron beams being irradiated onto the sample. Consequently, 4D-STEM is more efficient in terms of dose, reducing the total electron dose and minimizing specimen damage caused by electron beam irradiation.
